# Unveiling urban governance diversity: Clustering cities based on mitigation actions

**DOI:** 10.1007/s13280-024-01991-z

**Published:** 2024-03-28

**Authors:** Sombol Mokhles, Kathryn Davidson, Michele Acuto

**Affiliations:** 1 Faculty of Architecture, Building and Planning, University of Melbourne, Building 133, Masson Rd, Parkville, Melbourne, VIC 3052 Australia; 2 Melbourne Centre for Cities, University of Melbourne, Melbourne, VIC Australia

**Keywords:** A “more global” urban comparison, Finance and implementation, K-means clustering, Mitigation actions, Patterns, Urban climate governance

## Abstract

Embracing a “more global” urban comparison in scientific assessments of climate actions by cities is essential to drive greater and more inclusive participation in global efforts to curb climate change. This comparison needs to engage cities irrespective of their size and status: when we do so, distinctive patterns of urban climate mitigation actions across a diverse range of cities emerge. Employing K-means clustering as a pattern recognition method, this study compares cities based on selected aspects of their reported mitigation actions to the Carbon Disclosure Project. It explores whether the identified clusters facilitate the comparison of a socio-spatially diverse range of cities. The study identifies five clusters within two themes, namely the nature and finance–implementation of actions, shedding light on shared and distinct governance aspects of mitigation actions by cities. Notably, the study underscores how governance patterns transcend city size and global status. These findings offer valuable insights for broadening the comparative imagination of cities and inter-city networking opportunities.

**Supplementary Information:**

The online version contains supplementary material available at 10.1007/s13280-024-01991-z.

## Introduction

The role of cities in tackling climate change is now well recognised in a wide variety of international fora. If the 2015 Paris Agreement has offered a key turning point (Castán Broto and Westman 2020; IPCC 2022) in recognising the centrality of cities in climate actions, the recent 2023 CoP28 Climate Summit has also put cities in the spotlight with a dedicated Local Climate Action Summit embedded for the first time in the UNFCCC proceedings. This is an appreciation now rooted not just in practice, but also in the scholarly literature, where urban climate governance as a sub-field has evolved around viewing cities as a necessary subnational category for addressing climate change (Smeds and Acuto 2018; Castán Broto and Westman 2020).

While numerous studies in urban climate governance have favoured qualitative approaches (Van der Heijden 2019) and focus on specific case studies (Betsill and Bulkeley 2006; Castán Broto 2017; Busch et al. 2018), recent scholarly trends advocate for a shift towards quantitative methods and large *n*-sample studies. This has been particularly the case through the growing purchase of “urban science” (Bettencourt 2021) approaches seeking to expand the scientific approach to urban issues through a variety of methodological experimentations. This move aims to facilitate a broader comparison of cities and expedite collective climate actions (Raven et al. 2019; Castán Broto and Westman 2020; Creutzig et al. 2020). However, within the current landscape of large *n*-sample comparative studies in urban climate governance, there is a notable deficiency in capturing diversity among cities. Many of these studies lean towards specific categories, like global cities or those in the Global North (Aguiar et al. 2018; Reckien et al. 2018; Abarca-Alvarez et al. 2019; Lee et al. 2020), often neglecting a more inclusive representation.

Other factors such as lack of data availability, particularly for less-resourced cities in the Global South, contribute to this bias (Jabareen 2023). Additionally, the prevalent use of performance-based metrics (e.g. emission reduction, see Hughes et al. (2019)) and hierarchical methods such as ranking and benchmarking have been criticised for perpetuating uneven representations (Kitchin et al. 2015; Robin 2021). These approaches often result in league tables of leaders and followers and are in turn potentially sidelining smaller, less economically internationalised cities (Leffel and Acuto 2018). This has resulted in a gap in understanding the unique challenges and contributions of diverse cities, requiring urgent attention (Van der Heijden 2019; Robin and Castán Broto 2021), which is all but compounded by a dearth of knowledge concerning the diversity of climate governance of cities regarding finance and implementation in urban climate governance (Robin 2022).

The inherent limitations of prevalent large *n*-sample comparative studies in urban climate governance can bear negative consequences for practitioners in city networks, too, not just scholars. These networks, as formal organisations aimed at fostering collaboration between cities around the world often with an emphasis on knowledge exchange to accelerate climate action (Davidson et al. 2019), have faced criticism for their uneven networking practices. In many cases, larger and more economically powerful cities enjoy heightened visibility and recognition within these networks (Gordon 2020), often being promoted as leaders (Lee 2013; Lee and Van de Meene 2012). Furthermore, as networks also use data for large *n*-sample city comparison (Hughes et al. 2019; Prieur-Richard et al. 2019), cities rely on often skewed comparisons to identify their peers for networking (Haupt et al. 2021; Kamiński 2023). Therefore, biased or hierarchical representations of cities in large *n*-sample comparisons can further exacerbate an already uneven landscape of networking practices, potentially further marginalising smaller cities and those more peripherally located in economic status.

This is problematic because smaller cities are at the heart of urbanisation trends. The majority of urban population growth (90%) anticipated before 2050 is expected to occur in cities with populations below 1 million inhabitants, particularly in the Global South, and predominantly in Asia and Africa (UN-DESA 2018). While large and more internationalised cities might have more of a responsibility to address climate change due to their larger emissions (Castán Broto and Bulkeley 2013), it is imperative to diversify knowledge in urban climate governance beyond these global hubs (Van der Heijden 2019). This is because smaller cities lacking global economic status have different priorities and face different barriers compared to their larger and global counterparts (van der Heijden 2018), and limited knowledge of these cities and the diversity of climate actions can limit networking opportunities and their potential for tackling climate change comprehensively across the planet.

This is where this study comes in. We aim to enhance the understanding of diverse governance pathways for various cities by integrating what critical geographers have tagged as a “more global” urban comparison into a relational approach to data using pattern recognition methods. While Robinson (2016) introduced the concept of a “more global” urban comparison to challenge pre-determined city categories, no studies have deployed this viewpoint into quantitative analysis. The same can be said of applications of pattern recognition methods like clustering to urban climate action. This move, we posit, can expand the knowledge of diverse climate actions by cities. Pattern recognition methods, such as clustering, could allow for the identification of new configurations in data without imposing preconceived assumptions or a priori theories (as argued by Duminy and Parnell (2020)), although we recognise this is a normative “urban science” standpoint in itself (Acuto et al. 2018).

Further, instead of conventional quantitative metrics such as emissions and GDP per capita, this paper adopts governance aspects of urban climate action, such as sectors, finance, and implementation, as alternative starting points for city comparison, as highlighted in the literature (Castán Broto and Bulkeley 2013; Robin 2022). The purpose is to include diverse cities including smaller cities (below one million inhabitants) and their diverse climate actions. This implies an explicit effort, and thus to some degree in our view a postcolonial sensibility, that calls us to expand the case of cities considered beyond presumed global city status, for instance, those of the Global and World City (GaWC) network analysis (Global and World Cities Research Network 2020). Such sensibility enables opening up to “more global” urban climate action experiences that have received little attention to date.

To do so, we step beyond definitions of global cities, characterised by central roles in the global economy and as assessed through (GaWC) rankings, take what recent scholarship has called a “more global” urban approach, and apply this to comparison. This builds on Robinson’s (2022a) call for a “more global” urban theorising urging a move beyond conventional and hierarchical categories, promoting the comparison of a diverse range of cities. We do so building on our previous calls to open up more globally (meaning more inclusively) the bases of urban science, towards building a more diverse picture of global urban experience (Acuto et al. 2018)—in climate action in particular.

By investigating the patterns of cities’ governance aspects of mitigation actions based on a sample from the Carbon Disclosure Project (CDP), our approach facilitates comparison among different cities and finding heterogeneity of mitigation actions. This provides a pathway for cities to consider themselves in relation to a broader international context of “peers” and expand their networking opportunities.

## Conceptual framing for enriching diversity of urban climate actions with a more global urban science

### Unveiling the diversity of urban climate actions

Westman and Castan Broto (2022) have rightly critiqued the homogenisation of urban climate governance, by advocating for the exploration of alternatives and plurality in understanding urban climate governance. This for us entails tackling two key aspects of this homogeneity. Firstly, urban climate governance should account for the experience of diverse cities (Van der Heijden 2019; Robin and Castán Broto 2021), including smaller cities, without global city status. Secondly, we should recast and expand our examination of climate finance and implementation. Robin (2022) contends that the prevailing literature predominantly leans towards market-based finance for large infrastructure funding mechanisms, influenced by market logic. In response, Robin (2022) urges a better grasp of the heterogeneity of financial instruments, actors, and everyday practices. She highlights the potential of small-scale projects, such as decentralised renewable energy initiatives, which are often likely to better engage just climate actions compared to large infrastructural projects.

Previously, qualitative studies have been the main vehicle to explore the diversity of financial instruments at play in urban climate action. For instance, Bracking and Leffel (2021) delved into the role of states in supporting climate actions in the form of carbon credits and New Green Deals. Philanthropy has emerged as another funding mechanism, particularly within international city networks, critiqued for focusing on short-term, incremental change, and technical solutions, while emphasising economic co-benefits for tackling climate change (Papin and Beauregard 2023).

This is not to urge for a shift away from qualitative research. Qualitative investigations have enriched our understanding of cities’ climate actions in various urban contexts. For instance, Westman and Castán Broto (2018) scrutinised the implications of climate partnerships in Chinese cities, within a semi-authoritarian context. Leal and Paterson (2023) revealed how city networks tend to promote particular kinds of investment, coercing cities in the Global South, such as Lima and Mexico, to prioritise mitigation over adaptation, sidelining their urgent local needs. Similarly, Bigger and Millington (2020) demonstrated that instruments like green bonds, even when used for adaptation policies, can exacerbate the existing inequalities in cities and pose risks for the urban poor.

### The diversity of climate actions in large *n-*sample studies

Current investigations focused on greater inclusivity and heterogeneity in urban climate governance often lean towards qualitative approaches, relying in turn on few select case studies. How could we leverage large *n*-sample comparative studies to enhance such efforts? This section explores the insights derived from existing *n*-sample studies, exploring their main focus and their limits.

Large *n*-sample studies present mixed findings on the mitigation-adaptation dichotomy in urban climate policies. There is more evidence that cities’ mitigation actions are more prevalent than adaptation (Busch et al. 2018; Reckien et al. 2018), particularly in the Global North. Nonetheless, a recent study by Hsu and Rauber (2021) with a focus on a more diverse range of state and non-state actors (including cities, regions, countries, and companies) found a higher prevalence of adaptation policies in the national-level policies of developing countries compared to developed countries. Surprisingly, no significant difference in cities’ adaptation or mitigation policies was observed in developed and developing countries in this study.

Some large *n*-sample studies have revealed the diversity of cities’ climate actions in terms of topics or governance arrangements, including finance and implementation. Some have relied on cities’ climate initiatives to understand the properties of prevalent actions across cities, such as the type of actions and their finance and implementation. For instance, Castán Broto and Bulkeley (2013) showed the heterogeneous mix of actors, settings, and governance arrangements such as finance and implementation in urban climate governance across climate initiatives (experiments) of 100 global cities around the world, primarily concentrated on mitigation. They found that cities largely focus on urban infrastructure, built environment, and transport. Similarly, Palermo et al. (2020) noted that European cities (*n* = 315) emphasised building and transport in their mitigation policies, and municipalities’ self-governing policies.

Others have employed heuristic methods of pattern recognition to understand climate action patterns, in terms of aspects such as policy foci or topics and emission reductions across cities (Abarca-Alvarez et al. 2019; Lamb et al. 2019; Sethi et al. 2020). Lamb et al. (2019) employed a topic model as an unsupervised machine learning technique, identifying diverse topics and sectors in 4000 mitigation case studies, revealing regional patterns. Abarca-Alvarez et al. (2019) applied a self-organising map as an unsupervised machine learning technique and identified four patterns of governing adaptation best practices across European cities. Additionally, Hsu and Rauber (2021) utilised natural language processing (NLP) to identify 30 topics in state and non-state actors’ climate strategy documents, revealing their distinct focuses on specific sectors. Finally, Sethi et al. (2020) found the pattern of mitigation actions from 867 studies in terms of their GHG abatement potentials. This tells us large *n*-sample studies offer an exploration of cities’ climate actions or policies from mitigation-adaptation dynamics to intricate patterns in topics and governance arrangements.

Yet, despite the strides made by previous large *n*-sample studies, two critical limitations persist. Firstly, the majority of these studies fail to compare diverse cities, focusing primarily on global or European cities (Lamb et al. 2019; Hsu et al. 2020). Notably, bibliometric analyses, such as those by Lamb et al. (2019) and Sethi et al. (2020), rely on successful case studies based on biased search engines, such as Scopus and Web of Science. Therefore, they often tend to dismiss small- and medium-sized cities, especially in the Global South and regions such as Africa and Asia (Zhu and Liu 2020). Nonetheless, attempts are underway in large n-sample studies to become more inclusive in considering diverse cities. For instance, a recent study by Mokhles and Acuto (2024) has expanded urban climate imagination by reviewing reported mitigation actions to CDP across 800 local governments. They highlighted that using measures such as cities' sectoral focus and finance and implementation arrangements enables moving beyond cities' size and global city status.

Secondly, very few studies, such as that by Sachdeva et al. (2022), have utilised cities’ climate actions, case studies, or action plans for meaningful city comparisons. Case studies or cities’ climate actions reflect cities’ socio-cultural contexts, and they can be used for city comparison and finding city typologies (Lamb et al. 2019; Creutzig et al. 2020). Sachdeva et al. (2022) analysed 318 climate strategies or action plans of 315 cities with net-zero targets using NLP. While they presented an insight into the pattern of cities’ strategies based on the two constructed themes of ecology and infrastructure, their endeavour lacks a nuanced understanding of sectors and governance aspects of climate actions such as finance and implementation. We argue that employing pattern recognition methods could redress at least some of these two limitations.

### Enriching diversity of urban climate actions through a “more global” urban comparison

To overcome the limitations noted above, a “more global” urban comparison (Robinson 2022a), with an explicitly relational approach to data (Duminy and Parnell 2020), can be adopted. In our view, such comparison fosters an appreciation that urban innovation for climate can arise in all kinds of cities, with the potential to imagine their distinctive future (McCann 2004; Robinson 2006), while addressing perceived challenges of incommensurability in comparing different cities in different contexts (Robinson 2011b).

A “more global” urban comparison seeks to move beyond the traditional city divisions based on size or global economic status, encouraging comparison of diverse cities beyond their differences (Ward 2010; McFarlane and Robinson 2012; Robinson 2016, 2022a). One way to achieve “more global” urban comparisons is by comparing cities based on repeated features or generating alternative concepts for comparison (Robinson 2016, 2022b). This approach offers alternative starting points beyond size, development, and global city status, providing fresh insights into cities’ multiplicities (McFarlane 2010). For instance, Niranjana (2022) employed grounded empirical observations to compare the infrastructure-making processes of two water desalination plan investments in two coastal cities across different contexts (Chennai and London).

While critiqued for its limitation in revising urban theory due to limited case studies (Scott and Storper 2015; Storper and Scott 2016), a “more global” urban comparison has found applications in housing (Haas 2022), urban development (Robinson et al. 2022), regeneration (Teo 2022), and infrastructure (Niranjana 2022). Although the methods used are mainly qualitative, there are calls for more methodological innovation (Brill 2022) that lend themselves to the type of experiment we put in place in this paper.

Notably, this paper integrates a “more global” urban comparison into relational data approaches, a novel endeavour, advocated by Duminy and Parnell (2020), emphasising bottom-up, emergent processes in urban science. In this paradigm, hypotheses and insights are generated from the data rather than theory, fostering “guided knowledge discovery techniques” for pattern recognition (Kitchin 2016). Such approaches acknowledge their contradictions, political implications, and ethical assumptions at different stages of analysis and are open to critique (Duminy and Parnell 2020). Here, we utilise aspects of mitigation actions, like sectoral focus, finance, and implementation measures, as alternative starting points for city comparison informed by the debates summarised above in sections “Unveiling the diversity of urban climate actions” and “The diversity of climate actions in large n-sample studies”. This integration addresses a critical gap in large *n*-sample studies in urban climate governance, promising new ways for expanding networking opportunities between diverse cities.

## Materials and methods

Table 1 outlines the integration of a “more global” urban comparison into a relational approach to data-driven comparison at various stages of analysis to expand insights into the diversity of mitigation action across diverse cities. These stages encompass data collection, feature engineering, data pre-processing, and data analysis. Each step’s critical decisions based on the conceptual framework are presented in the table, with justifications elaborated in subsequent sections. All analytical procedures and spanning data collection to processing were executed using Python, leveraging different packages such as Pandas, Matplotlib, and Scikit-learn. This methodology ensures, in our view, a robust exploration of the diverse landscape of mitigation actions in cities, contributing to a nuanced understanding of urban responses to climate challenges.

**Table 1:** Key decisions at each analysis step for incorporating a “more global” urban comparison into a relational approach to data-driven comparison

Methodological approach		Key decisions
Incorporating a “more global” urban comparison into a relational approach to data-driven comparison	Analysis steps	Purpose: Expanding knowledge of diverse cities and their similarities and differences in their mitigation actions
	1- Data collection	• Selecting the CDP mitigation data set and examining the representation of diverse cities • Selecting the themes of comparisons based on cities’ governance of mitigation action but ensuring that they are available for diverse cities • Acknowledging the bias and limitations of data in both comparison themes
	2- Feature engineering and data pre-processing	• Organising the data for city comparison • Acknowledging the bias and limitations of data in deleting cities with missing or limited data
	3- Data analysis	• Selecting K-means as a non-hierarchical method • Paying attention to the often-overlooked cities in the interpretation of results
	4- Tests of association	• Using statistical tests to find whether there is a lack of association between the identified patterns of mitigation actions and cities’ size and global economic status

### First step: selection of the data set and fields of actions

#### Selection of the CDP dataset

To expand knowledge of diverse cities based on their climate actions, this study utilises Carbon Disclosure Project (CDP) dataset. CDP serves as an open repository, recording an extensive amount of annually reported climate-related data of cities and companies (CDP 2021). Encompassing cities’ reported mitigation and adaptation actions, emission reductions, and renewable energy targets, the CDP aims to facilitate monitoring cities’ strides in addressing climate change. This study focuses on mitigation actions, given their higher prevalence than adaptation actions (Busch et al. 2018; Reckien et al. 2018). Our initial assessment of the CDP data set also reveals more detailed reporting on mitigation actions than on adaptation actions.

We do acknowledge a lingering inclination of CDP towards Global North cities (Hsu and Rauber 2021), and its exclusion of cities not affiliated with city networks that report into it, whilst concerns about data quality through cities’ self-reporting persist. Critiques by scholars like Hughes et al. (2019) and Gordon (2016) question the privileging of specific types of knowledge, such as energy use or emissions, accessible primarily to resourceful cities (Hughes et al. 2019). Despite these limitations, previous studies have successfully employed CDP (Mokhles and Acuto 2024; Hsu and Rauber 2021; Sachdeva et al. 2022).

It is noteworthy that CDP captures the essential properties of cities’ climate actions, including sectors, finance, and implementation—the key aspects highlighted in the conceptual framework section “The diversity of climate actions in large n-sample studies”. These measures can offer innovative entry points for city comparisons that are urgently needed. As detailed in section “Bias and representation in the final data sets”, the subsequent analysis demonstrates that, despite these biases, these measures are available across a socio-spatially diverse range of cities, aligning with the prospects of a “more global” urban comparison.

#### Selection of fields of actions and themes of comparison

Four fields were chosen from those outlining cities’ sectors and mitigation implementation and finance, aligning with the conceptual framework 1.^1^ Additionally, ensuring inclusivity for diverse cities, including those often overlooked, was a paramount consideration, though we recognise and discuss the limitations in section “Bias and representation in the final data sets”. The chosen fields include:

- “Sector of actions”: Categorical values indicating the sector of cities reported actions within the ten predefined categories in CDP (e.g. building, water management, energy efficiency, and waste). Please refer to Supplementary Information for more information of the categories of sectors of mitigation actions.
- “Implementation status”: Ordinal values denoting whether the action has been implemented or is in the initial stages of scoping and pre-feasibility studies.
- “Finance status”: Ordinal values indicating the financial status of actions—whether secured or in the initial pre-feasibility and feasibility stages.
- “Primary fund source”: Categorical values, illustrating the action’s primary source of funds.

Two themes were defined based on these fields:

- Nature of actions: Combining actions’ sector of focus with their number of actions, akin to Castán Broto and Bulkeley’s (2013) second indicator on the types of action, or topics identified by Hsu and Rauber (2021) or Lamb et al. (2019). For the nature of actions, each city is represented as an eleven-feature vector. This vector captures the percentage of actions across ten sectors and the normalised value of the number of reported actions.
- Finance–implementation: Merging finance and implementation status with primary fund source in a nine-feature vector for each city. This vector consists of cities’ average implementation status, finance status of actions, and the percentage of actions funded by each of the seven primary funding sources. Despite the differences in measures, the rationale for this combination is its ability to describe how actions are governed. This theme closely aligns with Castán Broto and Bulkeley’s (2013) inquiry into the governance of initiatives, and Robin’s (2022) call for understanding the heterogeneity of urban climate governance. These themes not only facilitate broader city comparison but also enable an in-depth investigation into the nature of mitigation actions and their governance concerning finance and implementation.

### Second step: feature engineering and data pre-processing

The second step involved feature engineering and data pre-processing^2^ for clustering cities based on their actions, focusing on the selected fields of action. As per above, we reorganised the dataset and fields into two themes: the nature of actions and actions’ finance–implementation. Subsequently, four pre-processing steps were applied to each data set: summarising, quantifying and scaling, restructuring, and handling the missing data. Given this step led to the deletion of cities with missing or limited data, in line with the conceptual framework, biases, and limitations are presented after the feature engineering and pre-processing stage in section “Bias and representation in the final data sets”.

Principal component analysis (PCA) was deliberately excluded as a method for dimension reduction in this study. The rationale behind this decision is rooted in the study’s specific focus on the sector, implementation, and financial aspects of cities’ mitigation actions based on the conceptual framework. Unlike PCA, which identifies the combination of features as principal components, our approach prioritises the selected themes, facilitating a more straightforward interpretation of clustering results.

### Third step: data analysis using K-means clustering

The selection of K-means clustering as a heuristic, non-hierarchical, and relational comparison method (as presented in Table 1) aligns with the conceptual framework. Non-hierarchical methods such as clustering represent bottom-up and heuristic pattern recognition approaches (Kitchin 2016), allowing the identification of salient features (Rokach and Maimon 2005) of cities or distinct groups of similar cities. Although K-means was proposed more than 60 years ago, and many other clustering algorithms have been published since then, it is still one of the simplest and most popular partitioning clustering algorithms (Jain 2008).

K-means clustering was applied to the two restructured data sets based on the themes of the nature of actions and finance–implementation. The number of clusters was verified using an elbow diagram, determining that five clusters were appropriate for both themes of comparison.^3^ Subsequently, a five-by-five matrix was established by intersecting the two clustering approaches, including 225 cities in both clustering approaches. This matrix illustrates how the analysis expands knowledge of a diverse range of cities and the multiplicity of their actions while highlighting their commonalities. The matrix is presented in Sect. 3.4.

### Testing the identified patterns and the diversity of cities

Following the identification of groups of similar cities based on their nature of mitigation actions and finance–implementation arrangements, this paper explores whether the patterns are associated with cities’ size or global city status. To investigate this, an analysis of variance (ANOVA) test is employed to examine the association between clusters and cities’ population and global city status determined by the global connectivity index. ANOVA, as a parametric test, compares the mean square between samples to the mean square within the sample. It is suitable for assessing statistically significant differences in an outcome between more than two groups (Whatley 2022). It is particularly useful for examining the association between categorical and continuous variables. Given that clusters are categorical, the ANOVA test is applied to cities’ population size and global connectivity index as continuous variables. A significance level of 0.05 is chosen for the tests, with careful consideration of the requirements of the test, including normality and homogeneity of the variance, before confirming the association.

## RESULTS

### Bias and representation in the final data sets

Since this paper integrates a “more global” urban comparison into a relational approach to data, it is essential to report the final data sets’ properties and limitations. The pre-processing steps involved handling the missing and limited data. This section shows the final sample enables comparing diverse cities beyond their population and global city status, including smaller ones, without a global city status. So, we present the diversity of cities in terms of their region, population size, and global city status for both data sets of the nature of actions and finance–implementation.

Table 2 compares the representation of cities across different regions in the two data sets to the data on cities worldwide from the United Nations Department of Economic and Social Affairs (UNDESA 2018). It shows over-representation and under-representation of specific regions. We observe a significant over-representation of cities in North America (5 times UNDESA) and Europe (twice UNDESA). In contrast, East Asia has the highest under-representation in the two final data sets. Despite the dominance of North American and European cities (65%), the pre-processed data still contain 35% of cities in regions largely considered Global South.

**Table 2:** Comparing the percentage of cities in different regions in the UNDESA data set, the original CDP mitigation data set, and the pre-processed CDP mitigation data set

CDP regions		Africa (%)	East Asia (%)	Europe (%)	Latin America (%)	Middle East (%)	North America (%)	South and West Asia (%)	Southeast Asia and Oceania (%)
UNDESA		10	27	14	11	8	8	15	7
Original CDP dataset		7	2	27	27	1	30	1	5
Pre-processed dataset	Nature of actions	3	4	25	19	1	40	1	7
	Finance–implementation	4	5	26	19	1	37	1	7

As Tables 3 and 4 show, while on average, the cities’ population for both data sets is around one million, most of the cities’ population is below 300,000 (denoted by median). Moreover, the significant variation in cities’ populations (over 2 million) shows a diverse range of cities population-wise. The GDP per capita at the country level shows high average values because most cities (about 65%) in the data sets are from North America and Europe with wealthy countries. Nonetheless, the high standard deviation (22 412) indicates the significant differences across the countries and the presence of cities from low-income countries. We used cities’ global city ranking (based on GaWC (Drudder and Taylor 2016)) as a measure of cities’ engagement in the global economy to present a better picture of cities’ economic aspects than GDP per capita. Most cities do not have a global city status (around 67 per cent in the nature of actions and 64 per cent of cities in the finance–implementation data set).

**Table 3:** Summary statistics of population GDP per capita and Global city ranking of nature of actions data set

	Population	GDP per capita	GaWC (Drudder and Taylor 2016)
Count	285	281	94
Mean	1 060 849	46 701.3	174.89
std	2 084 474	22 412.7	136.4
Median	271 616	53 469	134
Min	1171	1338	1
Max	13 951 640	101 649	481

**Table 4:** Summary statistics of population GDP per capita and Global city ranking of finance–implementation of actions data set

	Population	GDP per capita	GaWC (Drudder and Taylor 2016)
Count	240	237	87
Mean	1 289 044	45 294.3	183
Std	2 651 814	22 563.6	139.6
Median	353 670	51 426	148
Min	4603	2770.7	1
Max	21 000 000	88 240.9	481

The high average ranking of the remaining cities indicates that they are not among highly global cities. So, we showed that despite the regional bias, the data set includes a diverse range of cities in terms of population and global city status. It also covers smaller cities (below one million population) with lower global city status.

### Patterns of cities based on the nature of actions

**Fig. 1:**
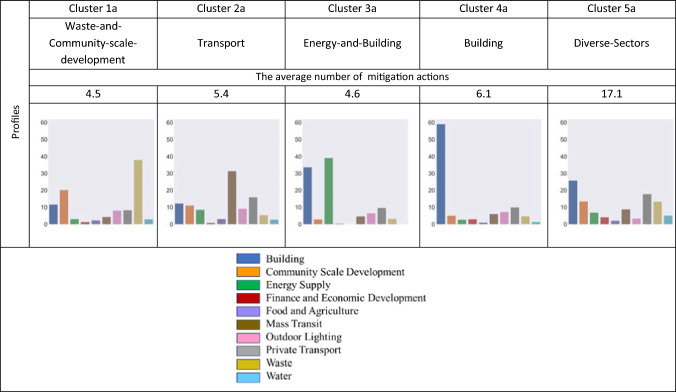
Clusters based on the nature of actions. Profiles indicate the average percentage of cities’ actions in each of the ten sectors

Utilising the first theme based on the nature of actions, five distinct clusters of cities (Clusters 1a–5a, as shown in Fig. 1) were identified. Each cluster of nature of actions includes the most similar cities based on sectoral combinations across ten sectors and the number of actions. Four out of five clusters (Clusters 1a, 3a, 4a, and 5a) focus on one or two specific sectors out of ten, denoting that these cities prioritise specific sectors compared to other sectors.

- Cluster 1a’s priority area is waste (40% of actions) and community-scale development (20% of actions).
- Cluster 2a focuses on transport since mass transit and private transport constitute around 45% of cities’ actions.
- Cluster 3a (32 cities) focuses on energy supply and building (with around 40% and 32% of actions, respectively).
- Cluster 4a emphasises the building sector much more than other sectors (on average, around 58% of cities’ actions).

Cluster 5a is the only cluster without a specific sectoral focus, as it has a relatively balanced distribution of actions across various sectors, denoting cities’ authorities across multiple sectors. This cluster, comprising 58 cities, has the highest average number of actions (17.1). In addition to having an average of 25% of their actions in the building sector, around 20% are in private transport, 15% are in waste and community-scale development, and less than 10% are in the remaining sectors.

Each cluster was assigned a label based on the nature of action profiles that reflects their sectoral combination (Fig. 1).

### Patterns of cities based on finance–implementation theme

**Fig. 2:**
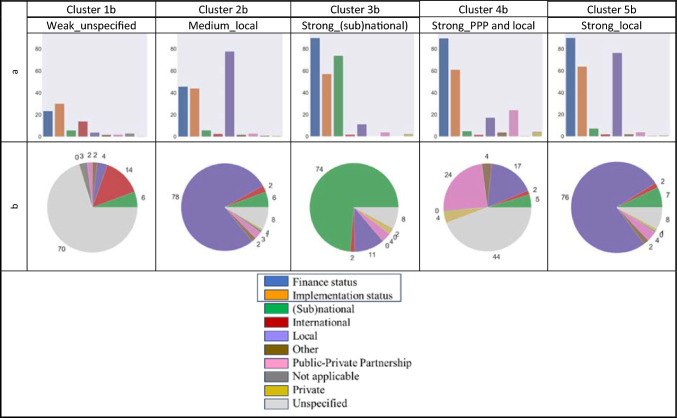
Clusters based on actions’ finance–implementation; **a** the first two bars on the left in each cluster diagram (blue and orange) show cities’ average value for finance status and implementation status in order. The following 6 bars refer to the percentage of actions funded by different sources; **b** pie charts for the average percentage of cities’ actions funded by different sources in each cluster

Similarly, utilising the second theme based on actions’ finance–implementation, five distinct groups of cities were identified, reflecting cities’ financial and implementation similarities and differences (Clusters 1b–5b). Figure 2 presents the clusters of finance–implementation.

Three out of five clusters (Clusters 3b, 4b, and 5b) demonstrate high finance and implementation status for their actions but distinct profiles for their source of funds. For instance, Cluster 5b shows high finance status (above 85%) and implementation status (around 60%), with considerable reliance on local sources (76% of actions) for 81 cities. Clusters 3b and 4b have equally high finance status and implementation status, but their priorities in funding are different. Cluster 3b focuses on (sub)national funds (74% of actions for only 17 cities), while funding sources for cities in Cluster 4b are distributed mainly between public–private partnerships (24% of actions for 57 cities) and local (17%), although the source of 44% of their actions is unspecified.

The remaining clusters (Clusters 1b and 2b) have relatively lower finance and implementation status. Cluster 1b (with 33 cities) shows the lowest finance status (around 20%) and implementation status (30%). Since the source of funds for 70% of cities’ actions is not specified, it shows that cities have been either less successful in encouraging different actors to fund their mitigation actions or face challenges in reporting their actions’ source of funds. International is the highest fund source (for only 14% of cities’ actions) for cities in this cluster. Cluster 2b, with 37 cities, has a medium financial and implementation status (around 40%). However, like Cluster 5b, the key fund source for Cluster 2b is local for 78% of their actions.

We interpreted the finance–implementation profiles of clusters based on their respective fields and assigned descriptive titles to each cluster. The first word acts as an adjective describing cities’ implementation and financial status of their actions, followed by their primary fund sources (Fig. 2).

### The multiplicity of cities’ nature of actions and finance–implementation

**Fig. 3:**
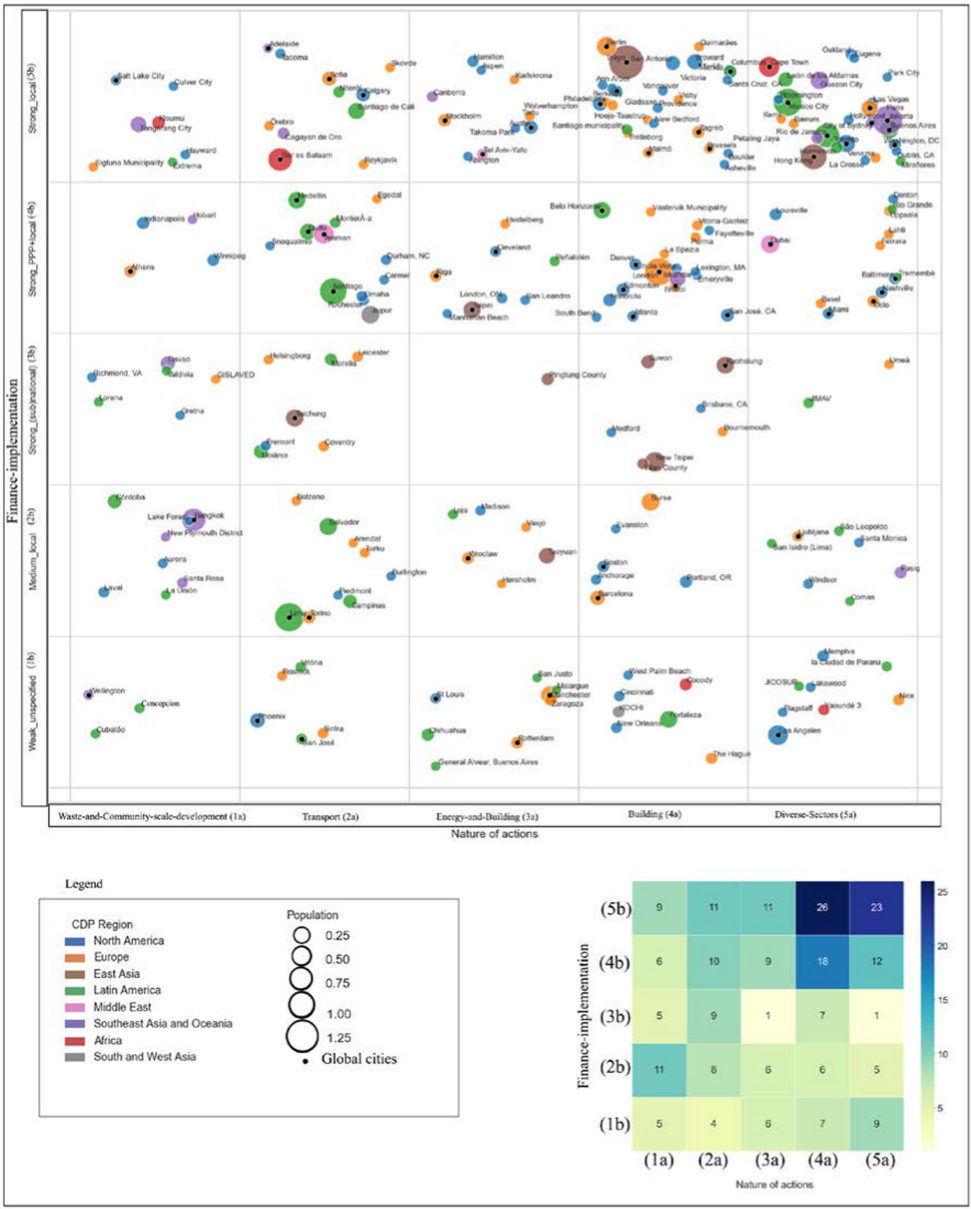
Matrix of cities’ typologies based on their nature of actions and finance–implementation considering cities’ global city status, population size, and regions. (The position of cities in each cell is randomly assigned for representation only.)

In order to provide a nuanced depiction of governance of mitigation actions based on both nature and finance–implementation, we constructed a five-by-five matrix at the intersection of the two comparison themes (Fig. 3). Each column represents one of the five clusters of the nature of actions, while each row shows one of the five finance–implementation clusters. The matrix incorporates cities’ size (represented by the circle’s size), global city status (indicated by an inner black dot), and regions (depicted in different colours), offering a comprehensive view of the range of cities.

The matrix reveals the governance patterns of cities’ mitigation actions across various cities. Out of 225 cities in Fig. 3, only about 64 have global city status, signifying that only 29% of cities are global, leaving about 71% without global city status. Furthermore, the matrix illustrates the prevalence of specific governance patterns among cities. For instance, most cities (26) fall into the intersection of Clusters 4a–5b, followed by 23 cities at 5a–5b, indicating the prevalence of local sources of funds (5b) for undertaking mitigation actions in building (4a) and across diverse sectors (5a). While some cells have more cities than others, there are cells with significantly fewer cities, such as 3a-3b and 5a-3b, suggesting a lower prevalence of cities relying on (sub)national funding sources. This finding indicates that we were able to reveal a diversity of responses to climate change based on our selected themes.

The matrix also indicates the prevalence of larger and global cities in some cells compared to others. Notably, 10 out of 23 cities in 5a–5b and eight out of 26 cities in 4a–5b are recognised as global cities. However, careful inspection of the matrix shows that most cells also include smaller cities, without a global city status. Even in the notable 5a–5b cell, which includes the most global cities (10 out of 23), 13 do not possess global city status. Furthermore, 14 of 23 cities in this cell have populations below one million. Thus, even within exceptional cells like 5a–5b, some cities are smaller, without global city status, highlighting their comparable potential to larger and global counterparts. It is worthwhile to note that specific cells like 5a–3b and 1a–3b predominantly include smaller cities, where none of the cities holds global city status. In other words, the matrix has enabled comparing smaller cities alongside their global and larger counterparts.

The matrix indicates internal diversity among different cities. Not only do we observe diversity of governance patterns in smaller cities, without a global city status, but large or global cities are dispersed across the different clusters. For instance, global cities such as Manchester, Los Angeles, Paris, Berlin, and London have very different profiles, as they are at different cells 3a–1b, 5a–1b, 5a–5b, 4a–5b, 4a–4b, respectively. Therefore, an initial insight from the matrix is that our clustering results have enabled comparison beyond cities’ size and global city status.

We further tested this hypothesis by exploring the association between size and clusters and global city status and clusters. Table 5 shows the results of ANOVA for both themes of the nature of actions and finance–implementation based on population and global connectivity index based on GaWC. The null hypothesis is that there is no association between clusters and one of the attributes (size or global connectivity index). The table shows the sum of square (“sum_sq”), the mean of square (“mean_sq”), degrees of freedom (“*df*”), *F *value, (“*F*”), *p *value (“PR(> *F*)”) for the main effect of clusters on cities’ population and global city status. The final column shows if the null hypothesis is rejected. The chosen significance level was 0.05, and since the p values are not less than 0.05, none of the null hypotheses are rejected.

**Table 5:** ANOVA results for the association between clusters and cities’ size and global city status

Themes	Attribute	*df*	sum_sq	mean_sq	*F*	PR(> *F*)	REJECT
Nature of actions	Global connectivity index	4	2.64E+09	6.60E+08	1.78E+00	1.33E−01	FALSE
	Population	4	3.01E+13	7.52E+12	1.75E+00	1.39E−01	FALSE
Finance–implementation	Global connectivity index	4	2.91E+09	7.27E+08	1.913841	0.108868	FALSE
	Population	4	7.83E+12	1.96E+12	0.274955	0.893969	FALSE

## Discussion

The association tests confirm the independence of both the nature of actions and finance–implementation clusters from size and global city status. In other words, cities of varying sizes, with and without a presence in established global city rankings, exhibit distinct patterns in governing their mitigation actions. Consequently, our approach transcends conventional city comparisons based on global economic status and population, revealing “the diversity of urban responses” (Robin and Castán Broto 2021, p. 870) for a wide array of cities (Robinson 2022b).

Extending prior studies (Mokhles and Acuto 2024; Castán Broto and Bulkeley 2013; Palermo et al. 2020) that highlight the prevalence of infrastructure and building-centric sectors of mitigation actions, our analysis sheds light on diverse cities, including those often overlooked, and their nature of actions’ similarities and differences. Similarly, finance–implementation clusters offer insights into the financial and implementation dimensions of mitigation actions by cities, extending earlier observations that mostly focused on local funding sources for specific cities, such as global (Castán Broto and Bulkeley 2013) or European cities (Palermo et al. 2020). Our study not only confirms, but also extends these observations for a more diverse array of cities, highlighting subtle differences and similarities in finance and implementation of actions, and diverse sectors beyond the ones that are commonly studied.

The finance–implementation clusters underscore the heterogeneity of financial instruments across cities and different levels of implementation, aligning with recent findings (Robin 2022). Some cities rely on local sources (2b, and 5b), while others prioritise (sub)national (3b), international (1b), or a combination of local and public–private partnerships (4b). Future studies could delve into just implications of these different instruments in climate actions in cities. Notably, our results highlight the co-occurrence of weak finance and implementation with a relatively high proportion of international funds, suggesting a potential challenge in international funds effectively implementing mitigation actions. This finding aligns with previous investigations into barriers to receiving international and private funds, especially in developing countries (Rahman and Ahmad 2016; White and Wahba 2019).

Despite the dominance of North American and European cities, the rows and columns of the matrix encompass cities from regions beyond the Global North, as indicated by different colours. Consequently, we have extended previous observations (Aguiar et al. 2018; Grafakos et al. 2018; Palermo et al. 2020; Reckien et al. 2018) by revealing the pattern of cities across different regions, including the Global South, which represents 35% of the data set. Much can be done to further expand this representation. We acknowledge the inherent bias of CDP data towards cities in the Global North. We also recognise its limitations in representing cities lacking resources to report actions or facing barriers to engaging in climate city networks, such as Chinese cities (as investigated by scholars such as Liu and Lo (2021)). To overcome this limitation, future studies should incorporate alternative and complementary data sets for comparative analyses.

The prevalent large *n*-sample city comparison tools raise concerns about reinforcing the uneven networking, positioning global cities from the Global North as leaders to be followed by others. These tools may exacerbate “the perceived performance gap” between wealthy top cities and smaller developing cities (Acuto et al. 2021, p. 369). Moreover, despite some signs of the inclusiveness of the overall system of networks (Cortes et al. 2022), scholars such as Kamiński (2023) have stressed the need for reimagining climate networking to encourage enabling possible futures for all cities. Our study, rooted in non-hierarchical city comparison, opens new pathways for networking beyond existing practices. City officials, informed by their cluster status, can consider networking with peers from their cluster or another cluster, fostering a more inclusive approach to learning and collaboration. The matrix offers a practical tool for expanding the comparative imagination of cities and encouraging more nuanced, context-aware networking practices.

One limitation is the cross-sectional focus on the CDP and ICLEI’s 2019 mitigation data set without considering the potential dynamics of knowledge exchange between cities. Future studies can incorporate a temporal analysis to better understand the potential learning links between cities. Additionally, longitudinal studies can track the evolution of urban climate actions, exploring the causal links between drivers (as found in Mokhles and Davidson 2021) and the change in urban climate actions.

This analysis can be further overlaid with the existing city networks and trajectories of cities on net-zero targets to evaluate the performance of city networks and climate actions of a diverse range of cities. Such analyses contribute to the calls (Robinson 2011a, 2016; Acuto and Rayner 2016; Acuto et al. 2021) to understand the processes of sustainable transition and climate actions, especially in cities that are often overlooked.

Our approach opens avenues for more in-depth qualitative analysis across different contexts, promoting questions about whether cities, including smaller cities without global city status, exhibit similar or different profiles to their larger and global counterparts (Fig. 3). For instance, the matrix shows Basel and Rio de Janeiro have similar nature of action profiles, as they have many actions in diverse sectors but different finance–implementation profiles. While Basel has a high implementation/finance status, with most actions funded mainly by a public–private partnership, Rio de Janeiro has relied on local fund sources. Questions could be raised on why Basel has primarily secured funding from a public–private partnership and local sources, while Rio de Janeiro mainly relied on its local funds. So, rather than being an end point to view “systematic regularities” (as argued by (Storper and Scott 2016)), our results offer a starting point for viewing the heterogeneity of cities in terms of their mitigation actions.

## Conclusion

This paper presented the different patterns of mitigation actions reported by a diverse range of cities in terms of nature and finance–implementation. By adopting a relational approach and integrating a “more global” urban comparison, we employed K-means clustering on CDP mitigation data (based on nature and finance–implementation) to enhance our understanding of mitigation patterns by cities. Despite data limitations, our analysis aimed at diversifying city comparisons beyond size and global city status, aligning with aspirations of a “more global” urban comparison.

Throughout the study, particular emphasis was placed on the inclusion of diverse cities, especially smaller ones without global economic status, in the data collection, processing, analysis, and interpretations. This approach offered novel insights into the various pathways of mitigation actions for a broad range of cities, as confirmed by the association tests. Despite the limitations, our study successfully unveiled the heterogeneity of mitigation actions of cities, encompassing diverse cities, including those often overlooked, with most cities in the final data sets having populations below one million and lacking global city status.

The results can be used to expand the comparative imagination of cities based on their mitigation actions in both research and practice beyond their size and global city status. Our study revealed the similarity and difference patterns among socio-spatially diverse cities based on their nature and finance–implementation of actions. We identified diverse patterns of mitigation actions by cities that were not associated with their size and global city status. Therefore, our findings indicated that the focus of urban climate governance on larger and global cities is not justified and smaller cities without global city status undertake mitigation actions across different sectors, with varying finance and implementation arrangements. Future studies can further investigate the profiles of the often-overlooked cities and how they govern their mitigation actions.

Additionally, from a practical point of view, our clustering results presented an opportunity to reimagine networking practices between cities. Moving away from prevalent uneven practices that primarily highlight larger cities with global city status as sources of information, our approach fostered peer-to-peer learning between cities, while highlighting the potential of cities that are smaller and without global city status. Future studies can further investigate the practical implications of our results in climate city networks and how they can inform the existing practices in established networks such as C40 and ICLEI.

Although our paper focused on climate mitigation actions by cities, we believe our conceptual framing can have wider applications. Large *n*-sample studies can be integrated with a “more global” urban comparison to understand the heterogeneity of cities in other areas, such as adaptation actions, housing, poverty reduction, and health. Future research can explore different ways that a “more global” urban comparison can be incorporated into a relational approach to data to enable the unveiling of urban heterogeneity in other realms of urban scholarship.

### Supplementary Information

Below is the link to the electronic supplementary material.

Supplementary file 1 (PDF 764 KB)

## Data Availability

Codes and data to reproduce the figures are available on figshare (10.26188/20114921.v1). The original data set of CDP’s mitigation action was accessed in 6th Oct-2020, as part of the publicly available CDP repository.

## Footnotes

1 For an overview of CDP dataset and its fields of actions, please refer to Section 1 and Section 2, Figure S1 and Table S1 in Supplementary Information.

2 More information in Supplementary Information, Section 4, Table S3 and Figure S2.

3 For details of cluster numbers and their justification, please refer to Supplementary Information, Section 5 and Figure S3.

## Code availability

All the feature engineering, data pre-processing, and clustering steps were conducted in Python (Version 3.7.6), using Jupyter Notebook. Packages used for pre-processing and feature engineering include Pandas and Numpy. The packages used for plotting were Matplotlib, Seaborn, and Sklearn was used for clustering. We used KelbowVisualizer from the yellowbrick.cluster to find the elbow diagrams for each clustering approach. We also used adjustText and textwrap to edit some of the plots.
