# Supplementary material for: Unveiling urban governance diversity: Clustering cities based on mitigation actions
Source: Ambio. 2024 Mar 28;53(8):1152–67. doi: 10.1007/s13280-024-01991-z (PMC11183020; doi:10.1007/s13280-024-01991-z)
Supplement: Supplementary file 1 — (PDF 764 KB) [file 13280_2024_1991_MOESM1_ESM.pdf]

Ambio

Supplementary Information

This supplementary information has not been peer reviewed.

Title: **Unveiling Urban Governance Diversity: Clustering Cities Based on Mitigation Actions**

Contents of supplementary

1. An overview of the data set..3
2. The fields of actions in the CDP original data set..3
3. Detail of sector of actions..4
4. Detail of feature engineering and pre-processing..5
5. Details of clustering method and validation of cluster numbers..8

Table of figures and tables

Table S1- Fields of actions in the CDP data set and fields selected for clustering..3
Table S2-Detail of subfields under the main fields of cities' sectors of mitigation actions..4
Table S3- Processed fields of CDP mitigation actions 2019 for the record of actions..6

Figure S1- Representation of tabular data features and terminology..3
Figure S2- Examples of the restructuring process of the two data sets: a- nature of actions, b- Finance-implementation..8
Figure S3- The elbow diagram for selecting the number of clusters based on distortion score for: a- the nature of actions (top: distortion, bottom: silhouette), b- finance-implementation (top: distortion, bottom: silhouette)..11

1. An overview of the data set

The 2019 CDP mitigation action data set is a tabular dataset, which means it is in the format of a table that consists of rows and columns. Figure S1 shows the features of a tabular dataset and their respective terminology, each of which will be further discussed for clarity and consistency across the thesis. Rows or records are horizontal features of a tabular dataset. In the case of the 2019 CDP mitigation actions dataset, each row includes the details about each reported mitigation action. Columns are vertical features, which are usually identified by a name, which is known as a field. In the case of the 2019 CDP mitigation dataset, each field refers to an aspect or feature of mitigation actions. A value is at the intersection of a column and a row (or record). In the case of the CDP mitigation dataset, values describe the state of action concerning a specific field.

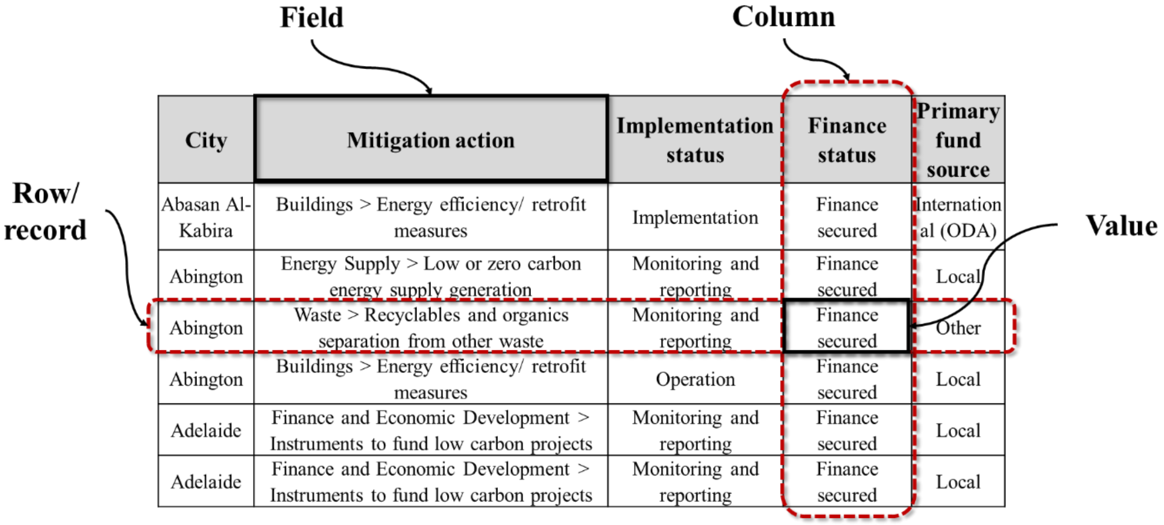

Figure S1- Representation of tabular data features and terminology

2. The fields of actions in the CDP original data set

The 2019 CDP mitigation actions data set included around 3779 rows of reported actions across more than 815 account numbers, which are unique numbers assigned to mainly organisation entities that report to CDP. Among these 815 account numbers, 587 had available values for the city name, covering 2740 actions. The CDP's 2019 mitigation actions data set consists of cities' reported actions for reducing their emissions in 2019. It is tabular data with 27 fields, of which 14 directly describe the actions' properties, such as the sector of actions and implementation details.

Table S1- Fields of actions in the CDP data set and fields selected for clustering

Fields	Fields describing actions	Selected for clustering
Year Reported to CDP	No	No
Account Number	No	No
Organization	No	No

City	No	No
Country	No	No
CDP Region	No	No
Reporting Authority	No	No
Access	No	No
Mitigation action (sector)	Yes	Yes
Action title	Yes	No
Means of implementation	Yes	No
Implementation status	Yes	Yes
Estimated emissions reduction (metric tonnes CO2e)	Yes	No
Energy savings (MWh)	Yes	No
Renewable energy production (MWh)	Yes	No
Timescale of reduction/savings/energy production	Yes	No
Co-benefit area	Yes	No
Action description	Yes	No
Finance status	Yes	Yes
Total cost of project	Yes	No
Total cost provided by the local government	Yes	No
Primary fund source	Yes	Yes
Web link to action website	No	No
Population	No	No
Population Year	No	No
City Location	No	No
Last update	No	No

Since the data set contained sporadic missing values across all the fields, we focused on four fields of actions close to the climate governance indicators used by Castán Broto and Bulkeley (2013) in alignment with our conceptual framework. We deleted the fields that reduced the number of actions below 1700 and the number of cities below 240. So, the selected fields also ensure the representation of more cities. The four fields of focus out of 14 on cities' climate governance are sectors, finance status, implementation status and primary source of funds.

3. Detail of sector of actions

For more clarity on the sector of cities' mitigation actions, Table S2 presents the subfields or subsectors under each category of actions' sectors, as defined by CDP.

Table S2-Detail of subfields under the main fields of cities' sectors of mitigation actions

Mitigation action	Subcategory
Building	Energy efficiency/ retrofit measures
	Building performance rating and reporting
	Building codes and standards
	On-site renewable energy generation
	Switching to low-carbon fuels
	Carbon emissions reduction from industry
Community-Scale Development	Green space and/ or biodiversity preservation and expansion
	Building standards
	Eco-district development strategy
	Urban agriculture

	Transit oriented development
	Brownfield redevelopment programs
	Low carbon industrial zones
	Compact cities
Energy Supply	Transmission and distribution loss reduction
	Low or zero carbon energy supply generation
	Smart grid
	Optimize traditional power/ energy production
Finance and Economic Development	Instruments to fund low carbon projects
	Developing the green economy
	Low-carbon industrial zones
Food and Agriculture	Encourage sustainable food production and consumption
Mass Transit	Improve bus infrastructure, services, and operations
	Smart public transport
	Improve rail, metro, and tram infrastructure, services and operations
	Improve fuel economy and reduce CO2 from bus and/or light rail
Outdoor Lighting	LED / CFL / other luminaire technologies
	Smart lighting
Private Transport	Improve fuel economy and reduce CO2 from motorised vehicles
	Infrastructure for non-motorised transport
	Awareness and education for non-motorised transport
	Improve the operations of shipping ports
	Improve the efficiency of freight systems
	Improve fuel economy and reduce CO2 from aviation
Waste	Recycling or composting collections and/or facilities
	Waste prevention policies and programs
	Improve the efficiency of waste collection
	Recyclables and organics separation from other waste
	Landfill management
	Improve the efficiency of long-haul transport
Water	Wastewater to energy initiatives
	Water use efficiency projects
	Water recycling and reclamation

4. Detail of feature engineering and pre-processing

The second step of feature engineering and pre-processing follows four steps. Firstly, we summarised the values of fields with more than ten unique values to allow for an interpretable comparison (shown in Table S3). Secondly, we quantified the values using one-hot encoding or ordinal numbers and scaled the values using min-max normalisation in each field for both data sets. Thirdly, we restructured the two data sets to allow city comparison by calculating the mean value and combining multiple rows of actions that referred to a specific city (Figure S2). Fourthly, we handled the missing data by deleting cities without available aggregated values in their respective fields and cities with only one reported action. As a result, the nature

of the actions' data set covers 285 cities, and the finance-implementation data set includes 240 cities.

Table S3- Processed fields of CDP mitigation actions 2019 for the record of actions

**Themes**	**Main field**	**Original number/range of unique values**	**Number of summarised values**	**Summary values**	**Quantification method**	**Number and range of values after scaling**
Nature of actions	Sector/mitigation action	43	10<br>As described in 2	1- Building<br>2- Community-Scale Development<br>3- Energy Supply<br>4- Finance and Economic Development<br>5- Food and Agriculture<br>6- Mass Transit<br>7- Outdoor Lighting<br>8- Private Transport<br>9- Waste<br>10-Water	One hot encoding	2 values: 0 or 1
	Number of actions	1-48	Not applicable	Not applicable	Not applicable	Values between 0-1
Finance- implementation	Implementation status	7	7	1- Scoping<br>2- Pre-feasibility study<br>3- Pre-implementation<br>4- Implementation	Ordinal values	7 values between 0-1

				5- Implementation complete<br>6- Operation<br>7- Monitoring and reporting		
	Finance status	4	4	1- Pre-feasibility study status<br>2- Feasibility undertaken<br>3- Feasibility finalised, and finance partially secured<br>4- Finance secured	Ordinal values	4 values between 0-1
	Primary fund source	161	7	1- International<br>2- (Sub)national<br>3- Local<br>4- Public-private partnership<br>5- Private<br>6- Other<br>7- Not applicable	One hot encoding	2 values: 0 or 1

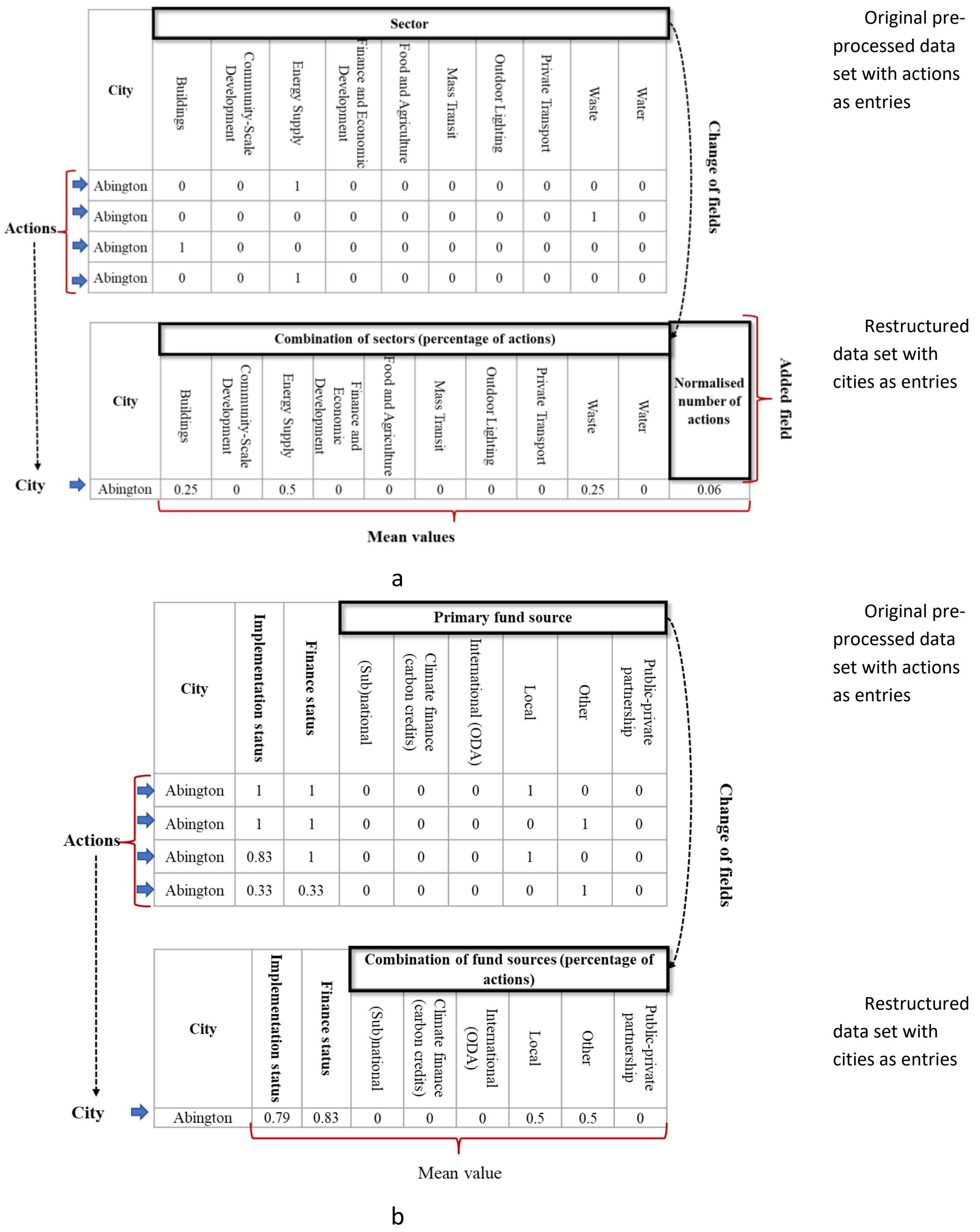

Figure S2- Examples of the restructuring process of the two data sets: a- nature of actions, b- Finance-implementation

5. Details of clustering method and validation of cluster numbers

We used the Scikit-learn package (Pedregosa et al., 2011) to apply the K-means algorithm, an unsupervised machine learning, to the two data sets to find the similarity between cities. We find distinct and interpretable clusters by dividing the data set into two groups that describe the nature of actions and implementation. The basic logic behind clustering is treating the

entities (in this case, cities) as points in the space with specific coordinates (based on their fields' values) and finding the points (cities) that are closer to one another. The K-means algorithm divides a set of N observations $\{x_1, x_2, \ldots, x_N\}$ into k sets of clusters so that the squared deviation of a cluster's members from the cluster's means ($\overline{C_j}$) is minimised through solving the following optimisation problem (Lloyd, 1982):

$$minimise \sum_{j=1}^{k} \sum_{i \in C_j} \left\| x_i - \overline{C_j} \right\|^2.$$

K-means operates as a hard-clustering technique, assigning each data point exclusively to a single cluster. Since the algorithm initialises cluster centres and iteratively updates the assignments of data points to centres, different initial points can yield different results. Consequently, a city might vacillate between clusters based on different initial points. To ensure the reliability of our clusters, we executed the algorithm 100 times, each with different random states for the initial point. Through this approach, we identified stable clusters, with minimal variation observed across 100 runs for both the nature of actions and finance-implementation themes.

Since the number of clusters is preassigned, we employed an elbow diagram to validate the appropriate number of clusters using two measures: distortion and silhouette score. The distortion score indicates the average distance between data points and their assigned centroid (Jolly, 2018). This score decreases as the members within clusters become more similar. Meanwhile, the silhouette score measures how well separated the clusters are, with higher scores reflecting more distinct clusters.

For the nature of actions, the elbow diagram suggests that 5 is an optimal value (as depicted in Figure S3). The distortion score significantly reduces when transitioning from 4 to 5 clusters, with no substantial decrease thereafter for nature of actions clusters. Similarly, the silhouette aligns with 5 as an appropriate number for nature of actions.

In the case of finance-implementation, the distortion elbow diagram also indicates five as the suitable number of clusters, even though it does not exhibit a significant decrease from 4 to 5 and a slowdown afterwards. The silhouette score for finance-implementation is three. To select the appropriate number of clusters for finance-implementation, clusters' profiles were for three and five clusters. While visualising three and five clusters were considered to determine the appropriate number of clusters for finance-implementation, we found that five clusters facilitated more meaningful distinction compared to three. Hence, we opted for five clusters for the finance-implementation theme.

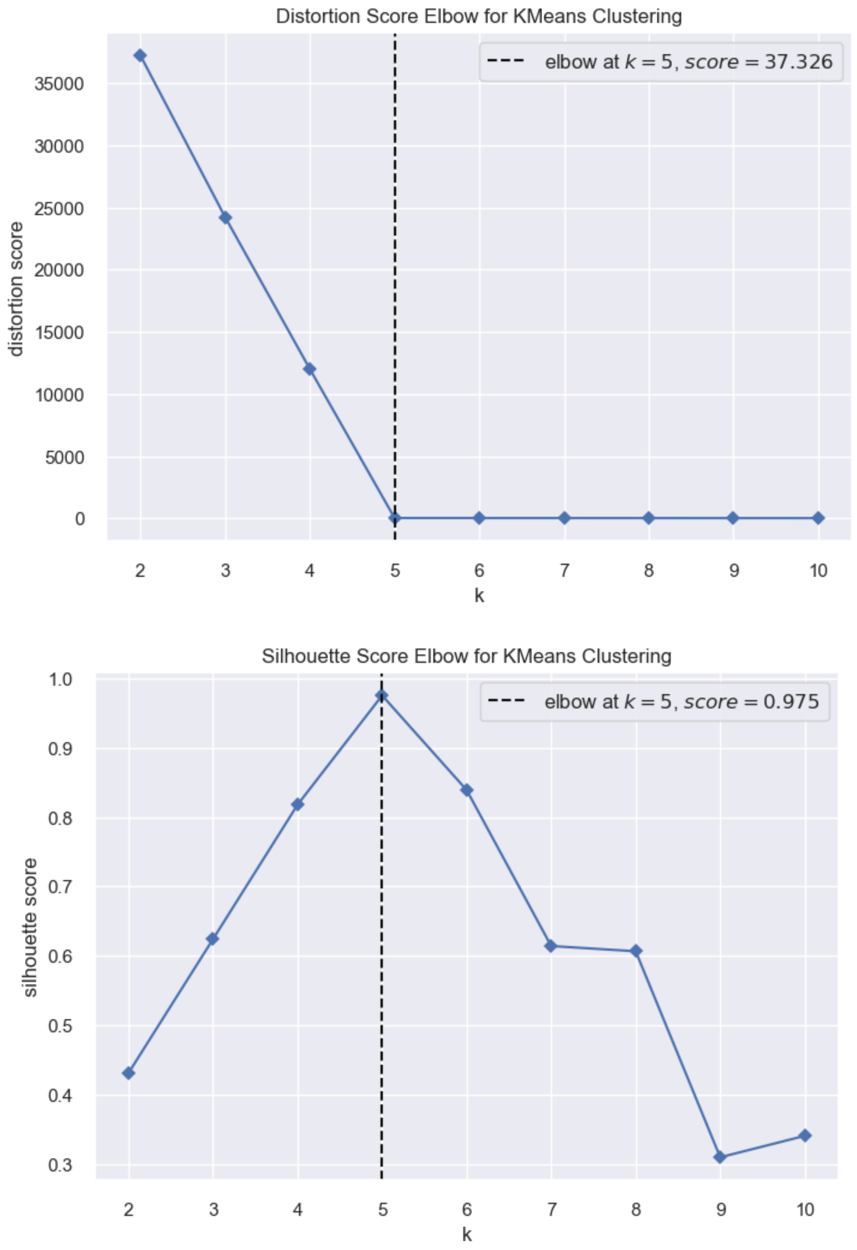
Distortion Score Elbow for KMeans Clustering
elbow at k = 5, score = 37.326
distortion score
35000
30000
25000
20000
15000
10000
5000
0
2
3
4
5
6
7
8
9
10
k
Silhouette Score Elbow for KMeans Clustering
elbow at k = 5, score = 0.975
silhouette score
1.0
0.9
0.8
0.7
0.6
0.5
0.4
0.3
2
3
4
5
6
7
8
9
10
k

a

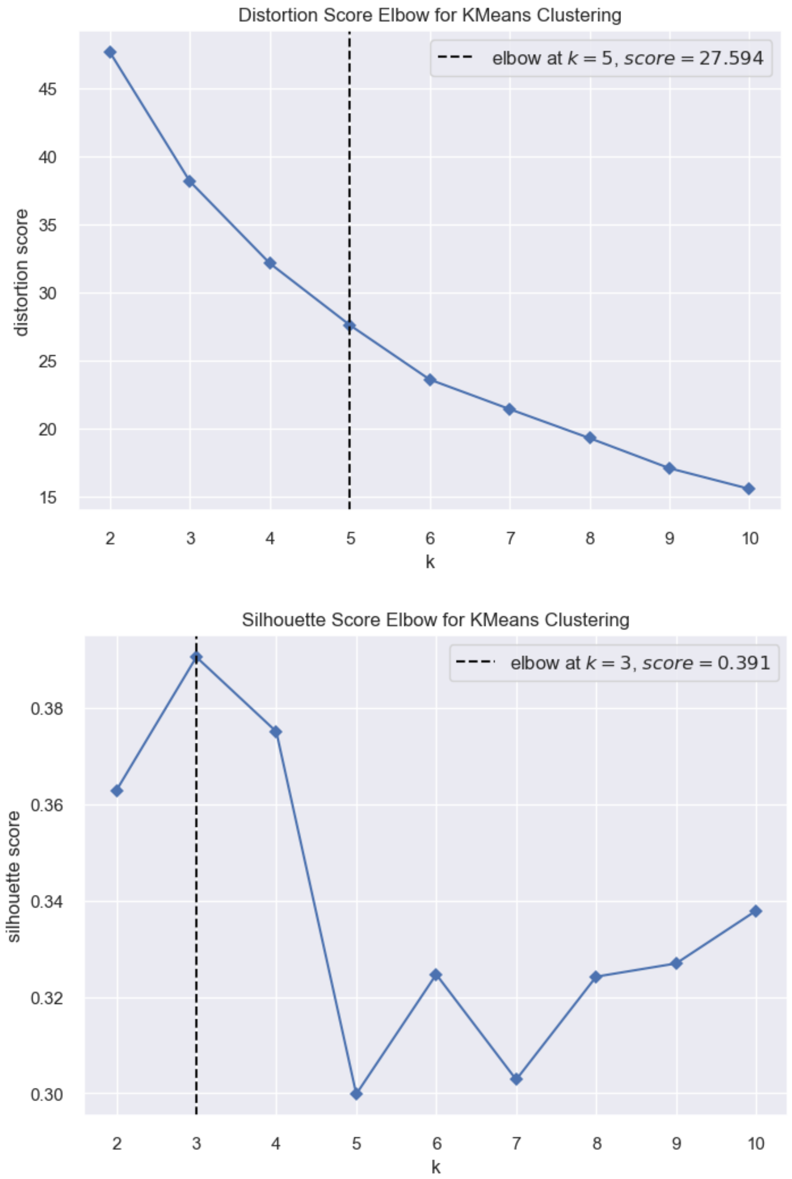

b

Figure S3- The elbow diagram for selecting the number of clusters based on distortion score for: a- the nature of actions (top: distortion, bottom: silhouette), b- finance-implementation (top: distortion, bottom: silhouette)
